# Different Length (DL) qPCR for Quantification of Cell Killing by UV-induced DNA Damage

**DOI:** 10.3390/ijerph7093376

**Published:** 2010-08-31

**Authors:** Knut Rudi, Irina Hagen, Bente Carina Johnsrud, Guro Skjefstad, Ingun Tryland

**Affiliations:** 1 Hedmark University College, Lærerskolealleen 1, 2418 Elverum, Norway; E-Mails: irinahagen@yahoo.no (I.H.); bcjohnsrud@hotmail.com (B.C.J.); gskjefstad@hotmail.com (J.S.); 2 NOFIMA Mat, Ås, Norway; 3 NIVA Norwegian Water Research Institute, Oslo, Norway; E-Mail: ingun.tryland@niva.no

**Keywords:** viable/dead cells, UV, quantitative PCR

## Abstract

We describe the different length (DL) qPCR method for quantification of UV induced DNA damage in cell killing. The principle of DL qPCR is that DNA damage inhibits PCR. Applications with different lengths can therefore be used to detect different levels of UV-induced DNA damage. The assay was evaluated on three strains of *Escherichia coli* exposed to varying levels of ultraviolet (UV) radiation. We show that DL qPCR sensitivity and reproducibility are within the range of practical application to detect the effect of UV cell killing.

## 1. Introduction

UV-treatment finds wide application for water treatment [1]. Unlike chlorination, UV does not leave chemical traces, and UV is also efficient against parasites [1]. The challenge with UV, however, is that it can be difficult to measure and quantify the decontamination effect. Although the DNA is lethally damaged by UV, the cells may physiologically behave as alive with both metabolic activity and intact cell membranes for a long period after UV exposure [2].

The aim of the work presented here was to utilize the property that damaged DNA inhibits PCR [3], and that different lengths of the PCR affect the likelihood of a DNA damage encounter [4,5]. Short PCR amplicons will have a lower likelihood of encounter than long PCR amplicons [6]. We wanted to test if the ratio between long (∼1,500 nt), medium (∼500 nt) and short (∼100 nt) PCRs can be used to measure the degree of DNA damage in a cell. We used *Escherichia coli* as a model in these investigations. *E. coli* is both a common water contamination problem and an indicator organism [7]. We present results showing that different length (DL) qPCR can detect lethal UV damage, and that the approach is promising as a tool for screening the effect of UV treatment.

## 2. Experimental Section

We evaluated the laboratory strain *E. coli* DH 5α, in addition to two *E. coli* strains (HIAS strain 1 and 14) isolated from the HIAS sewage treatment plant (Hamar, Norway). The HIAS strains were isolated using growth at 44.5 °C as a selection criterion, and the strains were confirmed as *E. coli* using a quantitative PCR test [8].

We used a mercury lamp with a major wavelength output at 254 nm for the UV treatment. Pure DNA and bacterial cells were UV treated following the same scheme. DNA or cells were added to sterile water to a concentration corresponding to approximately 10^6^ cells/mL. Fifty mL of the spiked water was added to Petri dishes, and exposed to UV irradiation at room temperature with the doses described in Table 1. Three parallel 1 mL samples were collected at each time point in black microcentrifuge tubes to prevent photoreactivation. The cells were harvested by centrifugation at 13,000 rpm for 5 min in a microcentrifuge, while the water containing pure DNA was not treated further. We used Prepman® Ultra for DNA purification with the protocol recommended by the manufacturer (Applied Biosystems, Foster City, CA, USA). Briefly, this protocol involves lysis by boiling and removal of PCR inhibitors by precipitation.

The DL qPCR amplifications were conducted in a 25 μL volume containing 1 × DyNAzyme II Hot Start-buffer, 2 μM each of forward and reverse primer, in addition to 1 μM Taqman-probe and 1 U DyNAzyme II Hot Start-enzym (Finnzymes, Espoo, Finnland). For pure DNA in water we used 5 μL template, while for bacterial cells 1 μL template were used. The reactions were run in an Applied Biosystems 7,500 Real-Time PCR System, using the software provided by the manufacturer for data retrieval (Applied Biosystems, Foster City, CA, USA).

The amplification efficiencies were determined by triplicate dilution series from 10^−1^ to 10^−4^ for each amplicon used using the calibration curve method [9] with the formula; PCR efficiency = 10^−1/slope^ – 1. The slope was determined by plotting the log of the dilution as a linear function of the Cq value using Microsoft Excel (Redmond, WA, USA). The primer sequences, amplification parameters, amplification efficiencies and reproducibility are presented in Table 2. The 16S rRNA gene universal probe described by [10] was used in all the q PCRs.

The amount of qPCR amplifiable DNA in each sample were determined by use of the the respective calibration curves for the amplicons used. The Cq values were used as input in the formulas, with the amount of amplifiable DNA relative to the standard curves as the output. Finally, for a given amplicon the effect of the UV treatment on amplificable DNA was determined by the difference in log of the estimated amount between two time-point (log amount time 2 minus log amount time 1).

Statistical analyses of differences in qPCR detectable DNA were done by a two-sample T-test for the biological replicates. All statistical tests were done using the TIBCO Spotfire S+ software (TIBCO, Somerville, MA, USA).

## 3. Results and Discussion

Despite the relatively low amplification efficiencies the amplicons used have a relatively high quantitative accuracy, as determined by the R2 values for the calibration curves (Table 2). The low and variable amplification efficiencies, however, preclude the direct comparisons of Cq values. We therefore chose to use the calibration curve transformed data for comparisons of the amount of qPCR detectable DNA. This was done by determining the corresponding dilution from the calibration curve for each Cq value. Since the dilution series were the same for all amplicons, the estimated amount of DNA can be compared across amplicons.

**Figure 1.**
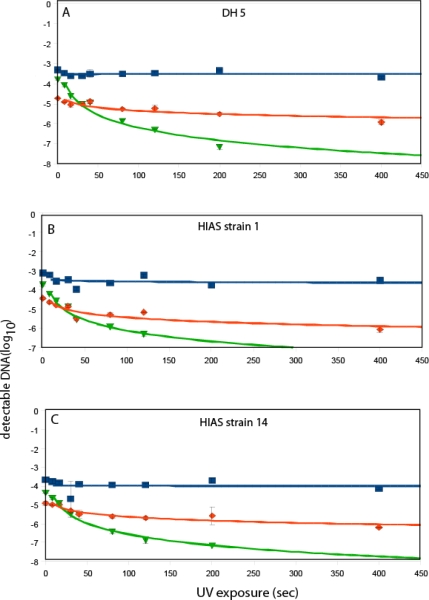
Time-series for DL qPCR of for UV treated *E. coli* DH 5α (A), HIAS strain 1 (B) and HIAS strain 14 (C). The blue curve show the short PCR, the red curve the medium PCR and the green curve the long PCR. Error bars show the standard deviation for three independent analyses of the same UV treated water. The scaling of the y-axis is relative to the respective standard curves.

UV-treated pure DNA showed a more than two log reduction in detectable DNA compared before UV treatment already after 16 sec exposure for the long DL qPCR. For the medium PCR fragment 40 sec UV exposure led to one log reduction in detectable DNA, while 400 sec was needed for same reduction for the short PCR. For the intact bacterial cells, all three strains showed approximately the same DL Q PCR response to the UV treatment (Figure 1).

For the long PCR there was a significant reduction in detectable DNA already after 8 sec (p < 0.05, t-tests), while for the medium PCR the reduction was not significant until 40 sec (p < 0.05, t-tests). For the short PCR, on the other hand, no significant reduction in PCR signal could be detected even after 400 sec (p > 0.05, t-tests). The plate counts showed a 4.5 log cfu reduction after 8 seconds of UV exposure for the laboratory strain DH 5α, while the HIAS strains showed only two log reduction after 8 sec. For the other time points analyzed no cfu’s were detected. By comparison to the cfu before UV treatment we found that cfu reduction was >5 log).

We were able to detect the effect of UV doses as low as 8 mWs/cm^2^ (8 sec exposure in Table 1) using DF qPCR. In Norway the current recommended UV treatment dose for water decontamination is 40 mWs/cm^2^ (www.fhi.no), so DL qPCR should be within that range of sensitivity for practical application. Although we have only evaluated DF qPCR for *E. coli* the assay can also be evaluated for other bacterial species since we use conserved 16S rRNA gene primer regions.

Interestingly, although all three strains tested responded similarly with respect to UV-induced DNA, there seemed to be a difference in survival, with the laboratory strain having the lowest survival. Thus, a potential further application of DL qPCR is to optimize UV treatment regimes [1].

## Figures and Tables

**Table 1.** Time and UV dose used for each technical replicate in the in the water treatment.

**Sample #**	**Time (s)**	**UV dose (mWs/cm^2^)**
1	0	not treated
2	8	8
3	16	16
4	30	30 *
5	40	40 **
6	80	80
7	120	120
8	200	200
9	400	400

* old recommended dose

** new recommended dose (www.fhi.no)

**Table 2.** Properties of the amplicons used.

**Amplicons**			**Thermocycling**			**Amplification**	
**Name**	**Primer sequence**	Position ^*^	**Denaturation**	**Annealing**	**Synthesis**	**Efficiency**	**R^2^**
Short F	GAAGAAGCACCGGCTAAC	529	95 °C – 30s	50 °C – 30s	72 °C – 30s	0.43	1
Short R	GCT TTACGCCCAGTCATTC	611					

Medium F	TCCTACGGGAGGCAGCAGT	375	95 °C – 30s	63 °C – 30s	72 °C – 45s	0.68	1
Medium R	GGACTACCAGGGTATCTAATCCTGTT	841					

Long F	AAGAGTTTGATCATGGCTCA	42	95 °C – 30s	55° C – 30s	72 °C – 90s	0.54	0.98
Long R	CGGTTACCTTGTTACGACTT	1546					

* Position relative to 5’ of the primers with respect to *E. coli* 16S rRNA.
